# The Insane in Chains: Literary Image of Russian Fiction and Historical Truth of the First Half of the 19th Century

**DOI:** 10.1192/j.eurpsy.2025.1851

**Published:** 2025-08-26

**Authors:** S. V. Motov, V. V. Motov

**Affiliations:** 1 Department of Slavic Languages & Literatures, University of Illinois Urbana-Champaign , Urbana, IL, United States; 2 Psychiatry, Social House “Stupino” (The Institution for Chronic Mental Patients with Disability) of The Department of Labor and Social Protection of the Population of the City of Moscow, Stupino, Russian Federation

## Abstract

**Introduction:**

A compatriot who is declared insane and ends up chained in a mental institution is a new and unexpected character that appeared in Russian fiction during its heyday in the first half of the 19th century. The theme of “madness” followed by “chaining” is repeated in the influential works of Alexander Pushkin, Alexander Griboyedov, Alexander Voeikov and other outstanding writers of this period.

**Objectives:**

Find out: (1) How historically accurate was this persistent artistic image — was it merely a literary convention or a true reflection of the status quo? (2) Was the shackling of patients “the standard of care” in psychiatric institutions in Russia and Europe in the late 18th and first half of the 19th centuries?

**Methods:**

A historiographical and comparative analysis was conducted, which allowed us to compare historical evidence and manuals on mental illness published in Europe in the period 1782-1845, as well as Russian professional literature on the history of psychiatry in Russia.

**Results:**

The artistic image of the “madman on a chain” largely corresponded to reality; moreover, in a number of cases, the horror of reality exceeded the artistic image.

**Conclusions:**

By bringing the image of the patient in chains to the forefront, Russian fiction attracted public attention to the topic, which was one of the factors that contributed to the opening of a significant number of new psychiatric hospitals in Russia in the second half of the 19th century, with a more humane attitude towards psychiatric patients.

**Disclosure of Interest:**

None Declared

